# Behind the pathology of macrophage-associated demyelination in inflammatory neuropathies: demyelinating Schwann cells

**DOI:** 10.1007/s00018-019-03431-8

**Published:** 2019-12-28

**Authors:** Hwan Tae Park, Young Hee Kim, Kyung Eun Lee, Jong Kuk Kim

**Affiliations:** 1 Peripheral Neuropathy Research Center (PNRC), Dong-A University College of Medicine, Busan, 49201 South Korea; 2 Department of Molecular Neuroscience, Dong-A University College of Medicine, Busan, 49201 Republic of Korea; 3 Advanced Analysis Center, Korea Institute of Science and Technology, Hwarangno 14-gil 5, Seongbuk-gu, Seoul, 02792 South Korea; 4 Department of Neurology, Dong-A University College of Medicine, Busan, 49201 South Korea

**Keywords:** Inflammatory demyelination, Wallerian degeneration, Paranodal demyelination, Schwann cell reprogramming, Myelin uncompaction, Axonal injury

## Abstract

In inflammatory peripheral demyelinating disorders, demyelination represents segmental demyelination in which the myelin sheath of a myelinating Schwann cell (SC) is completely removed by macrophages or a partial myelin degeneration in the paranode occurring due to autoantibodies attacking the node/paranode. For the segmental demyelination from living myelin-forming SCs, macrophages infiltrate within the endoneurium and insinuate between myelin lamellae and the cytoplasm of SCs, and the myelin is then removed via phagocytosis. During the macrophage invasion into the SC cytoplasm from the node of Ranvier and internodal areas, the attacked SCs do not remain quiescent but transdifferentiate into inflammatory demyelinating SCs (iDSCs), which exhibit unique demyelination pathologies, such as myelin uncompaction from Schmidt-Lanterman incisures with myelin lamellae degeneration. The longitudinal extension of this self-myelin clearance process of iDSCs into the nodal region is associated with the degeneration of nodal microvilli and paranodal loops, which provides a potential locus for macrophage infiltration. In addition to the nodal intrusion, macrophages appear to be able to invade fenestrated internodal plasma membrane or the degenerated outer mesaxon of iDSC. These SC demyelination morphologies indicate that the SC reprogramming to iDSCs may be a prerequisite for macrophage-mediated inflammatory demyelination. In contrast, paranodal demyelination caused by autoantibodies to nodal/paranodal antigens does not result in iDSC-dependent macrophage infiltration and subsequent segmental demyelination. In the context of inflammatory demyelination, the novel perspective of iDSCs provides an important viewpoint to understand the pathophysiology of demyelinating peripheral neuropathies and establish diagnostic and therapeutic strategies.

## Introduction of demyelinating Schwann cells

Axonal injuries result in the destruction of the myelin sheath and axon in the distal portion of the lesion sites via the process of Wallerian degeneration (WD). Peripheral nerves efficiently regenerate following axonal injury, but the central nervous system does not. One of the reasons for the regeneration ability of peripheral nerves after injury is associated with the rapid clearance of degenerating axon-myelin debris, which is performed by the cooperative interaction of myelinating Schwann cells (mSCs), a peripheral glial cell that synthesizes the myelin sheath, and macrophages, which infiltrate from the blood after injury [1, 2]. It has been shown that mSCs dedifferentiate into immature SCs, which no longer express myelin genes, such as Krox20 and myelin protein zero (MPZ), but re-express genes related to immature SCs, including p75 neurotrophin receptor (p75), during WD [3, 4]. In addition to these phenotype changes, the dedifferentiated mSCs exhibit unique characteristics that are not shown in immature SCs [1]. For example, the expression of lipolytic enzymes, autophagy induction for myelin digestion and secretion, and the secretion of inflammatory cytokines occur in the mSCs following nerve injury, which implicate the distinctive ability of mSC reprogramming for self-myelin removal [1, 3, 4]. Therefore, the transformed or dedifferentiated mSCs in WD are not just immature SCs but specialized for myelin clearance, and thus the cells were recently designated as “demyelinating SCs” (DSCs) [1].

**Fig. 1:**
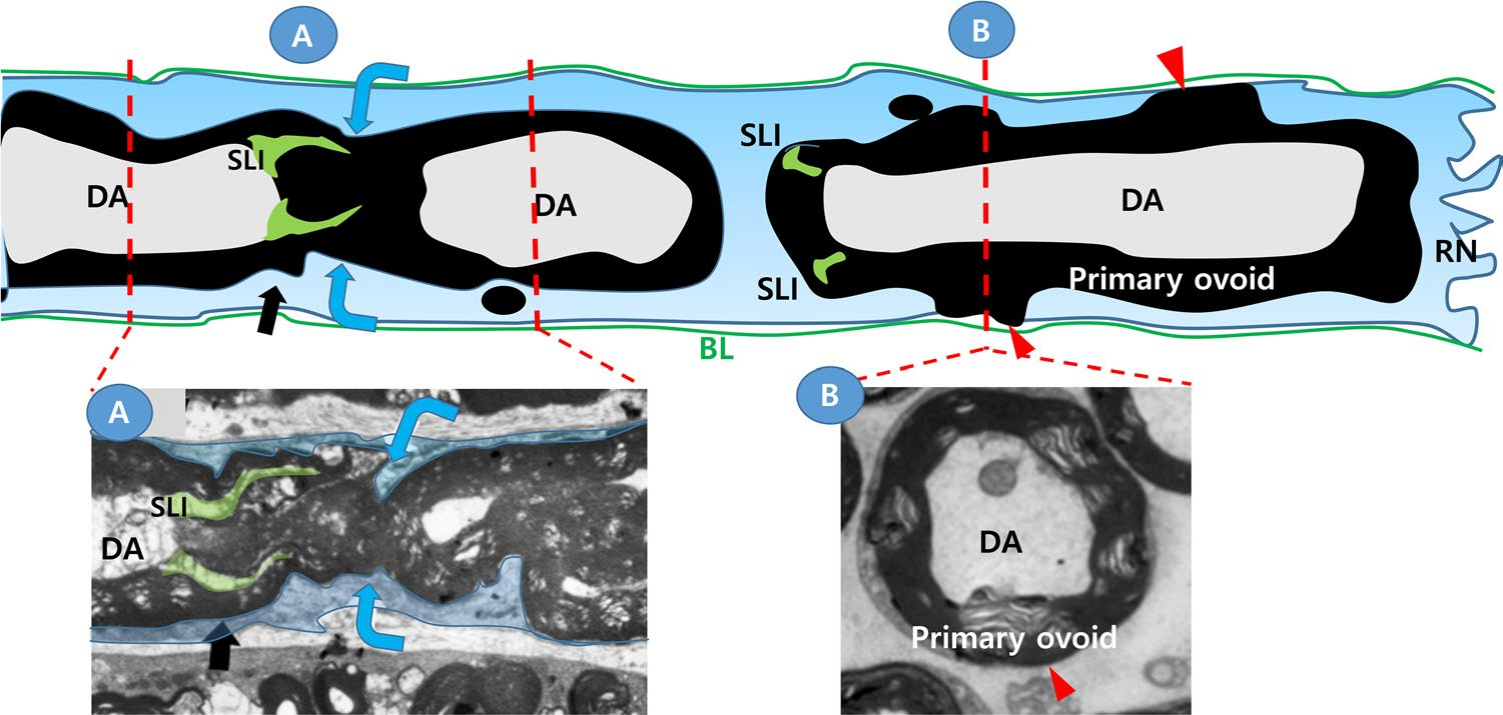
Schwann cell demyelinating processes in Wallerian degeneration. Upper panel; Schematic longitudinal drawing shows macrophage-independent demyelination processes by a demyelinating Schwann cell after nerve injury. Blue arrows; the location of myelin fragmentation adjacent to the Schmidt-Laterman incisures (SLI), red arrowheads; an area where the myelin sheath directly exposes to basal lamina of the endoneurium. Note that SLI is incorporated into one side of primary myelin ovoid after fragmentation. *DA* degenerating axon, *PO* primary ovoid, *RN* the node of Ranvier, *MV* microvilli, *BL* basal lamina. Lower panels; electron microscopic (EM) images of longitudinal (A) and cross (B) sections of demyelinating Schwann cells after injury. **a** Longitudinal EM image of the myelin sheath around the SLI [5]. Blue arrows indicate the location of myelin fragmentation. Black arrows indicate the SLI. **b** Cross EM image of the myelin sheath in the middle of a primary myelin ovoid [12]. At the node of Ranvier (RN), the paranodal loops are disappeared and the paranodal myelin layers fused each other to close the chamber of the myelin ovoid

Myelin clearance by DSC during WD is accomplished via the following sequential processes (Fig. 1). Firstly, the internodal compact myelin sheath is fragmented, which generates numerous myelin ovoids (or chamber) within the cytoplasm of DSC [1, 5–9]. The fragmentation site is not randomly determined, but it always occurs around the Schmidt-Lanterman incisures (SLI), which is a non-compact cytoplasm-containing area in the internode, and the SLI becomes incorporated into one side of the myelin ovoid after the fragmentation completes (Fig. 1). During this early stage of WD, the axonal cytoskeleton and the domain architectures of the myelin sheath are lost, and the primary myelin ovoid resides within the cytoplasm of DSCs (Fig. 1). Myelin fragmentation represents the cleavage and repair, or reunion, of all layers of compact myelin at the fragmentation site to close the chamber, which was demonstrated in previous studies [7–9] and Fig. 1. When the axon degeneration proceeds in a node of Ranvier, the paranodal axo-glial interaction dissolves, and the paranodal loops fuse with each other to close the chamber of the corresponding myelin ovoid (Fig. 1). Secondly, after the primary myelin ovoid is formed, a small myelinosome or secondary ovoid is generated from the primary ovoid, which is then digested by SCs via autophagy or exposed to the outside of the DSCs independently or together with the primary ovoid (Fig. 1b) [1, 10–12]. While this SC self-destruction progresses for 3–7 days after injury, the infiltrated macrophages invade into the endoneurial tube and engulf the primary or secondary myelin ovoids that were exposed outside the DSCs.

Inflammatory peripheral demyelination generally indicates partial or functional demyelination in the nodal/paranodal region due to the autoantibodies against the antigens present in these areas and a complete myelin loss in an internode, called segmental demyelination, via the demyelinating attacks of macrophages [13, 14]. Several biochemical features of DSCs in WD (wDSCs), such as the expression of c-jun and p75, together with shutdown of the expression of myelin genes and an increase in autolysosomes were also observed in the SC of the animal models of inflammatory demyelinating neuropathy, in which the axon is relatively intact [1, 10, 12]. Previous results and our recent study demonstrated the presence of potential DSCs in human inflammatory demyelinating nerves using biopsy specimens and patient sera, respectively [15, 16], which suggests a pathological implication of DSCs in demyelinating neuropathies. However, the contribution of the demyelinating ability of DSCs, which may be inevitably accompanied by these inflammatory attack factors, on the pathogenesis of inflammatory demyelination has not been significantly considered in the clinical field. Therefore, the present article reviews the emerging concept of an inflammatory DSC (iDSC) to investigate how the transformed SCs contribute to macrophage-dependent or -independent demyelination in inflammatory demyelinating neuropathies.

## Inflammatory demyelination by macrophages

Various conditions cause peripheral neuroinflammatory demyelinating diseases, which are considered primarily autoimmune diseases [14]. Daily activities are severely limited in most patients due to paralysis and sensory abnormalities. Guillain–Barre syndrome (GBS) is an acute inflammatory peripheral neuropathy, and acute inflammatory demyelinating polyradiculoneuropathy (AIDP) is a typical demyelinating form of GBS. Autoantibodies to the junctional proteins in the nodal/paranodal regions, such as neurofascin, were found in some of patient sera, but the pathological mechanisms of AIDP are not clearly elucidated in most cases [17, 18]. Neuropathic symptoms persist for more than 2 months in the chronic form of inflammatory demyelinating polyradiculoneuropathy (CIDP), and various clinical features and therapeutic responses to immunosuppressive agents may indicate the presence of a number of pathogenic mechanisms in CIDP development [13, 19]. It has long been suggested that the pathological demyelination in typical AIDP/CIDP is caused by cell-mediated immunity, which is provoked by autoantibodies to the SC membrane or myelin proteins, and as a result of this autoimmune reaction, inflammatory cells, such as macrophages, are mobilized to the peripheral nerves and execute demyelination [19–23]. Experimental autoimmune or allergic neuritis (EAN), which develops after the immunization to myelin proteins, such as P2 and MPZ, has been used as a good model of AIDP, and the inhibition of macrophage infiltration significantly suppresses the development of EAN [22, 24]. Macrophage-associated demyelinating pathologies in both inflammatory peripheral neuropathy models (EAN/B7-2 knockout non-obese diabetic mice) [10, 24–26] and patient biopsy specimens [21, 27] support the central role of macrophage-associated demyelination (MAD) as the cellular mechanism by which complete demyelination in an internode occurs in the classical inflammatory demyelinating neuropathy.

MAD is a pathological mechanism that relies on macrophage infiltration into the tube of the basement membrane that surrounds inflammatory nerves in the endoneurium. Macrophages within the endoneurium insinuate between the SC cytoplasm and myelin lamellae to phagocytose and completely remove the myelin [20, 21, 24–27]. Demyelination in MAD may be initiated via secretory lipases/proteases produced by macrophages [28], however it should finally involve the direct contact of macrophages with SC myelin within the SC cytoplasm to phagocytose the myelin [20, 21, 24–27]. Considering the remarkable plasticity of mSCs in various demyelinating conditions [1], there may be a contribution of DSCs to MAD. Therefore, we propose the potential intercellular mechanisms by which macrophage processes invade into the potential space between the SC cytoplasm and partially degenerated myelin lamellae in the node of Ranvier and into the internode during inflammatory demyelination.

## Unique characteristics of inflammatory demyelinating Schwann cells

**Fig. 2:**
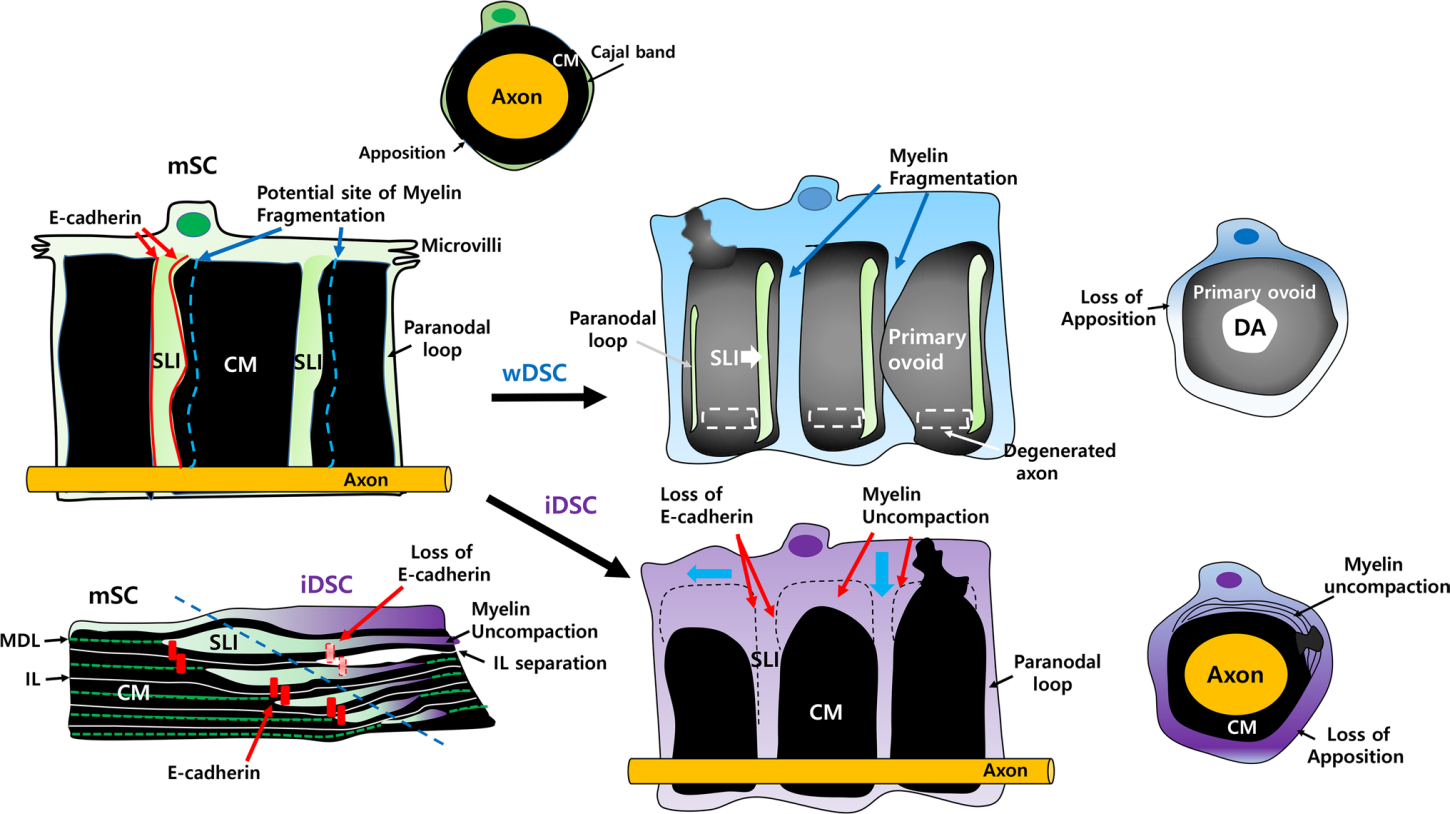
Unwrapped images of the myelin sheath of myelinating and demyelinating Schwann cells. The cytoplasm of myelinating Schwann cells (mSC) normally exists in the SLI, paranodal loops, and Cajal band. *CM* compact myelin. SLI; Schmidt-Lanterman Incisures, Blue dots; potential location of myelin fragmentation in Wallerian degeneration. Red dots; E-cadherin (adherens junction) in SLI. After axonal injury, the cytoplasm of Wallerian demyelinating Schwann cells (wDSC) increases and myelin fragmentation occurs near the SLI (along with blue dots in mSC), and the SLI is incorporated into one side of myelin ovoid. *DA* degenerated axon. In inflammatory demyelinating Schwann cells (iDSC), hypertrophic Schwann cell cytoplasm spreads into the SLI and paranodal loops (blue arrows). Loss of adherens junction in SLI may allow the the hypertrophic myelinolytic cytoplasm to dissolve myelin layers, resulting in the separation of the intraperiod line (IL). Myelin fragmentation (primary ovoid formation) could not occur due to an intact axon in iDSC. *MDL* major dense line

In contrast to wDSCs, iDSCs in inflammatory demyelination exhibit unique morphological features of myelin clearance that are not found in wDSCs due to the presence of an intact axon (Fig. 2, Table 1). The cytoplasm outside compact myelin of normal mSCs is divided into a characteristic cytoplasmic channel, the Cajal band, and an apposition, where the outer SC plasma membrane and outermost myelin lamella make a contact [29–31]. Both wDSC and iDSCs exhibit a hypertrophic cytoplasm with the loss of the typical pattern of cytoplasmic Cajal band and the apposition (Table 1, Fig. 2) [12]. In iDSC, the hypertrophic abaxonal cytoplasm was often associated with the separation of the major dense line (MDL) (Fig. 2), which is the potential cytoplasmic area between myelin lamellae, and the widening of intraperiod line (IL), the interposition of an extracellular face of adjacent myelin lamellae [26, 32, 33].

**Table 1:** Common and unique characteristics of inflammatory DSC compared to Wallerian DSC

Characteristics of DSC	Wallerian DSC	Inflammatory DSC	
Cytoplasmic hypertrophy	+	+	Evidence of DSC
Induction of c-jun/p75	+	+	Dedifferentiation markers
Autolysosome	+	+	An executor of myelin clearance
Myelinosome	+	+	Evidence of DSC
Loss of apposition	+	+	Related to cytoplasmic hypertrophy
Junctional protein destruction	+	+	General response of DSC
Paranodal retraction	+	+	General response of DSC
Myelin exocytosis (exposure to basal lamina)	+	+	Evidence of DSC
Myelin Fragmentation, Ovoid formation	+	−	Secondary to axonal degeneration
Ovoid incorporation of SLI	+	−	Secondary to axonal degeneration
Axon degeneration	+	−	Secondary to axonal injury
Myelin uncompaction	−	+	Related to myelin lamella degeneration
Intraperiod line separation	−	+	Related to myelin lamella degeneration
Outer mesaxon separation	−	+	Related to junctional region destruction

*DSC* demyelinating Schwann cell, *SLI* Schmidt-Lanterman Incisures

Myelin uncompaction has been found in various types of peripheral neuropathies [33]. Except the primary lack of myelin compaction due to the genetic abnormalities of myelin proteins such as MPZ [34, 35], most of peripheral neuropathies showing uncompacted myelin are associated with abnormal antibody production [33]. For example, polyneuropathy of POEMS syndrome, monoclonal gammopathy and anti-myelin associated glycoprotein neuropathy exhibited uncompacted myelin lamellae either inside or outer part of the myelin sheath [33, 36]. This finding may indicate that pathologic antibodies leaking into the myelin sheath through the mesaxon or nodal regions disrupt the compactness of the myelin [35]. Since anti-myelin autoantibodies are also implicated in the demyelinating form of GBS and CIDP, the myelin uncompaction observed in these inflammatory demyelinating neuropathies could be generated by the antibodies.

In normal compact myelin, the MDL and IL are maintained not only via the binding of the extracellular domain of myelin proteins but also via adherens junctional proteins, such as the E-cadherin complex, those are present at the intersection of the non-compact junctional regions (SLI/paranode/mesaxons) and compact myelin (Fig. 2) [5, 37–39]. In architectural point of view, the myelin uncompaction observed in various types of neuropathies was continuous with the paranode or SLI, that indicates the uncompaction might arise from the non-compact junctional regions where normal uncompaction is located [36]. Because the degeneration of junctional structures which can be represented by the loss of E-cadherin in the junctional regions is the common feature of DSC (Table 1) [5, 12], the reactive changes of physicochemical properties of the junctional regions by DSC may contribute to the initiation of myelin uncompaction by anti-myelin antibodies. As shown in Fig. 2, it is also likely that when the contents of hypertrophic cytoplasm of iDSC, which may contain autolysosomes or myelin-degenerating enzymes, migrate inward from the outer abaxonal side to adaxonal side, the flow goes through the SLI and paranodal loops, thereby contributing to the of separation of adjacent MDL and IL via initiation of destruction of the junctional structure.

Because the intimate contacts of each of the paranodal loops are also associated with the junctional proteins, including E-cadherin [38], the hypertrophic cytoplasmic flows toward the nodes of Ranvier would also alter the junctional structure in the paranodal loop, creating a swelling of the terminal loops and separation between loop layers. A previous electron microscopic study using sural nerve biopsy specimens from patients with anti-myelin-associated glycoprotein neuropathy, which show segmental demyelination, described a swelling of paranodal myelin terminal loops before they detached from the axolemma [40], which indicates the potential presence of iDSCs in this type of demyelinating neuropathy. In addition, another junctional structure, the outer mesaxon, appeared to be damaged by E-cadherin destruction in iDSC [12] and this may allow the entrance of antibodies into IL, further enhancing the demyelination changes by iDSC.

## Unique characteristics of inflammatory demyelinating Schwann cells may provide potential nodal routes for macrophage invasion

**Fig. 3:**
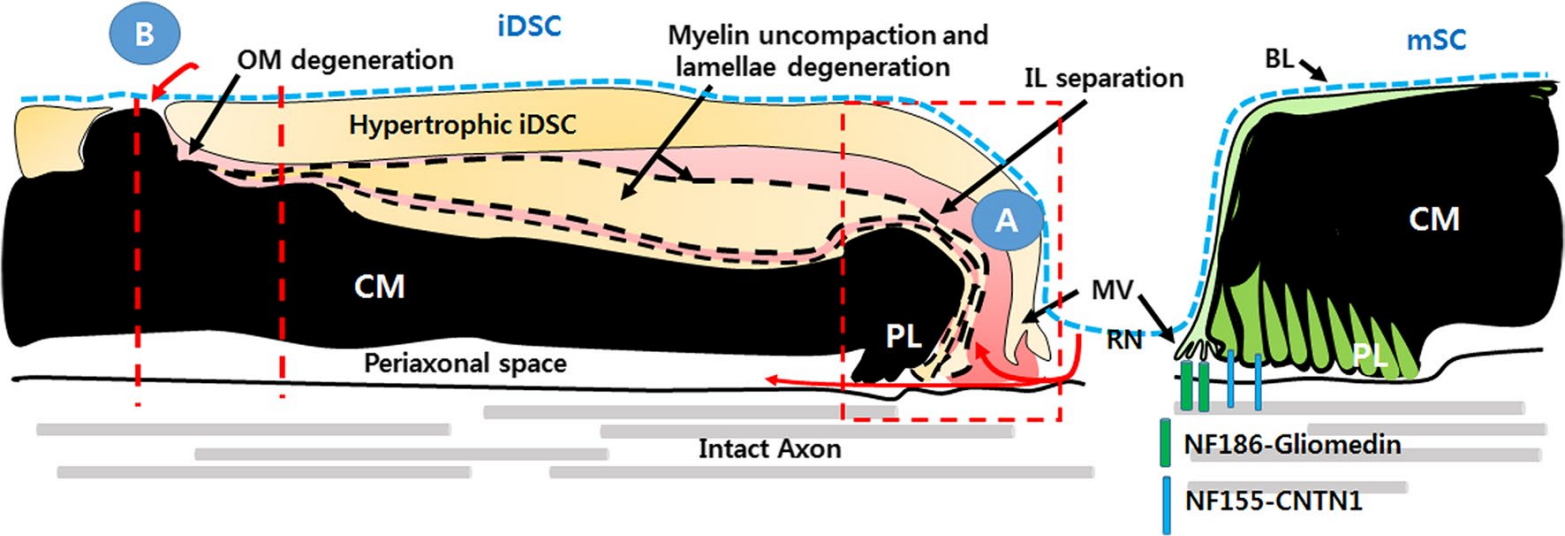
Schwann cell demyelinating processes in inflammatory demyelination. Schematic longitudinal drawing shows potential routes for macrophage invasion (red arrows). **a** Longitudinal extension of myelin uncompaction with intraperiod line separation from an internodal region to the node of Ranvier may disrupt nodal and/or few paranodal junctions, allowing macrophages to invade into the nodal area. **b** Internodal routes for macrophage invasion into the cytoplasm of inflammatory demyelinating Schwann cell (iDSC). The locus where degenerating myelinosome or the myelin sheath exposed to basal lamina (with a similar manner to Wallerian demyelination), or degenerated outer mesaxon (OM) may provide an internodal route for macrophage invasion. *CM* compact myelin, *PL* paranodal loop, *RN* the node of Ranvier, *MV* microvilli, *BL* basal lamina

Although many morphological studies have shown the removal of myelin by macrophages in inflammatory peripheral neuropathies, these studies have not provided information on the exact locus of macrophage penetration into SCs. Macrophage infiltration into the endoneurium removes myelin via a sparing of the SC, and the SC is a living cell while the myelin might be half-dead or exist as a myelin corpse [12, 23, 27]. Therefore, the possible entry sites of macrophages for myelin phagocytosis in the living SCs should be limited to where myelin lamellae are partially degenerated or separated from the SC cytoplasm. There might be few potential anatomical entrances in iDSCs for macrophages; between the SC microvilli and the first paranodal loop in the node of Ranvier or between the layers of paranodal loops (Fig. 3a); between the paranodal terminal loops and the axolemma (which extends to the periaxonal space) in the paranode (Fig. 3a); at external exposure site of the degenerating myelin in the internode (Fig. 3b); and at the degenerated outer mesaxon (Fig. 3b).

In a recent review, Koike et al. demonstrated the details of electron microscopic interpretations of macrophage inflow into the CIDP node region [23]. In the normal structure of the node of Ranvier, microvilli are abundant in the cytoplasm of the mSC at the nodal end, and several protein–protein interactions, such as NF185-gliomedin interaction, support the axon-to-SC microvilli interaction (Fig. 3) [38, 41, 42]. The next paranodal loops encircle the axon, and theoretically exhibit the same number of paranodal loops as the myelin lamellae (Fig. 3). Assuming macrophage infiltration in the node/paranode region, the nodal axo-glial interaction of the microvilli must first be destroyed, and the separation of the microvilli and the first paranodal terminal loop (first IL) would allow a macrophage to invade the space between the SC cytoplasm and outermost myelin layer (Fig. 3a). When the connection between the junction of the outer few paranodal terminal loops and corresponding paranodal axonal membrane is dismantled, macrophages may penetrate this region and insinuate into the space between the layer of the outer paranodal loops. On the other hand, when all of the paranodal axo-glial junctions are broken, macrophages may infiltrate into the periaxonal space. However, the final periaxonal invasion must be accompanied by the complete degeneration of every paranodal-axon interaction equivalent to the number of myelin layers, which presumes that more drastic changes of SCs are required for the periaxonal intrusion of macrophages.

According to previous electron microscopic studies of MAD, macrophage processes were primarily located between abaxonal SC cytoplasm and the outermost myelin lamella or in the separated IL of the outer myelin layers (red area in Fig. 3), rather than in the periaxonal space [12, 20, 21, 24–27]. The generation of these separated IL may be the result of physicochemical myelin degeneration by infiltrating macrophages, or a consequence of DSC changes with or without antibody attacks (Figs. 2 and 3), which means that the longitudinal flow of myelinolytic substances contained in the outer hypertrophic cytoplasm of DSCs to the node of Ranvier induces the degeneration of the nodal microvilli and outer paranodal loops (Fig. 3). This longitudinal separation of the IL and the inflow of macrophage infiltration are well documented in the longitudinal sections of electron microscopy of inflammatory demyelination [12, 23].

The above-mentioned morphological interpretations from the standpoint of SCs indicate that macrophage infiltration in the node region might require the characteristic demyelinating changes of mSCs, and it could not purely rely on the myelinolytic activity of macrophages or anti-myelin antibodies. Paranodal retraction was observed without antibodies against nodal/paranodal proteins in the EAN model, in which only the myelin protein P2 was used as an antigen [43]. Because the typical biochemical features of DSC are found in the EAN [12, 44], the transformed iDSCs may be the cellular mechanism of these nodal changes in EAN. Extensive paranodal demyelination was also observed in the segmental demyelination induced by lysophosphatidic acid and calcium ionophores, which are not related to the immune response [45, 46]. The neuropathy-associated with antibodies against myelin-associated glycoprotein, which is not a specific nodal/paranodal protein, showed paranodal abnormalities [40] that mimicked iDSC before segmental demyelination. Therefore, nodal/paranodal degeneration is not a specific pathology caused by autoantibodies against nodal/paranodal proteins, but it may be produced as the initial common outcome of segmental demyelinating processes. One of the mechanisms related to the myelin clearance in wDSC is actin polymerization, which induces myelin fragmentation and junctional destruction [5]. Because the main cytoskeletal structure of microvilli is actin filaments, SC actin polymerization may contribute to the degeneration of the nodal/axonal interaction in iDSC.

## Inflammatory demyelinating Schwann cells and nodo/paranodopathy

**Fig. 4:**
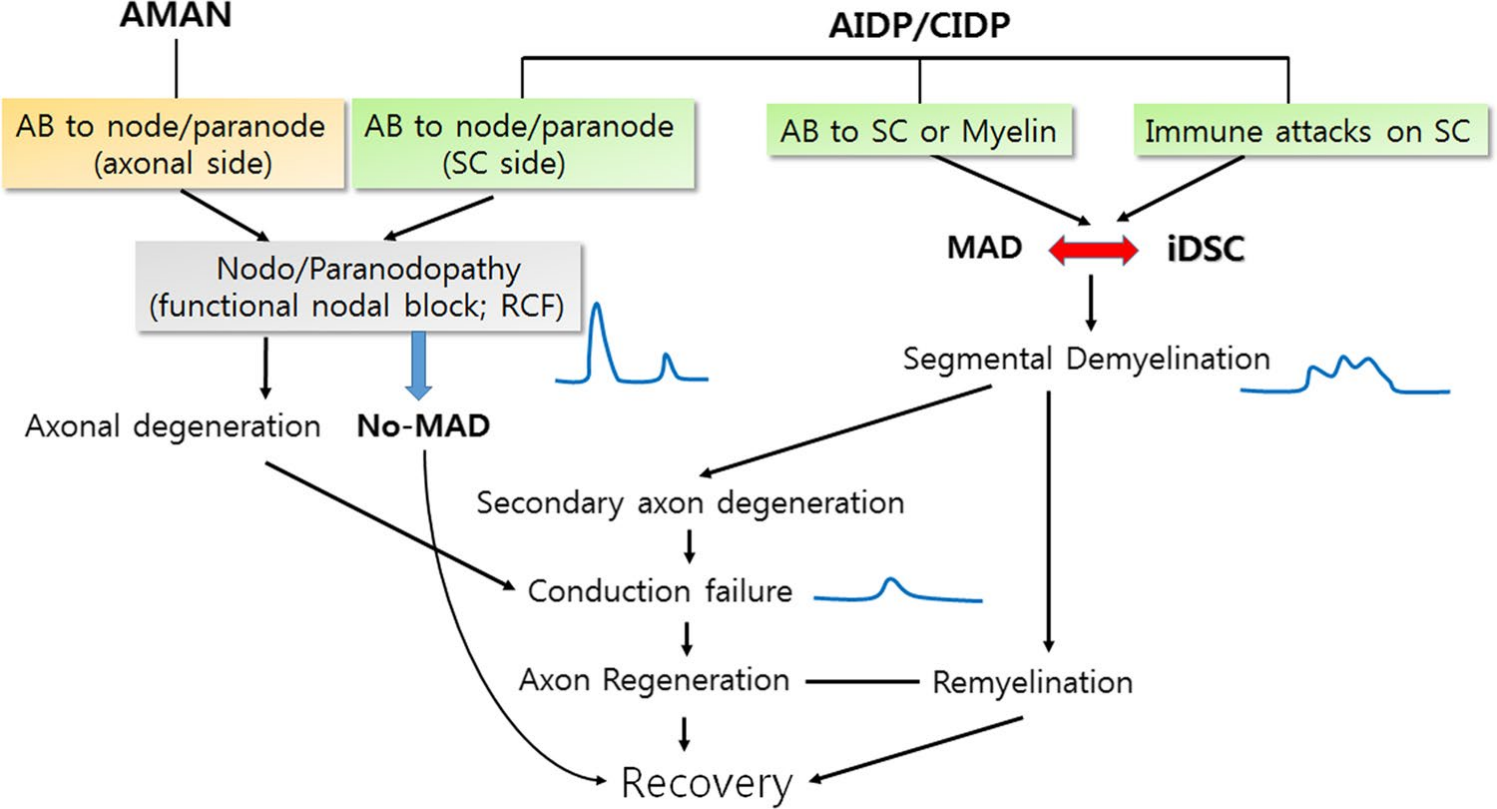
Clinical implication of inflammatory demyelinating Schwann cells (iDSC) with special references to paranodal and segmental demyelination. Segmental demyelination, which shows slowed conduction velocity and temporal spreading in electrophysiology, depends on the cooperative interaction of macrophage-associated demyelination (MAD) and iDSC. Specific antibodies to nodal/paranodal proteins are associated with paranodal demyelination (nodo/paranodopathy), and it is characterized by reversible conduction failure (RCF) in electrophysiology without MAD-iDSC interaction in pathomechanism. Even though paranodal demyelination is not sufficient for eliciting segmental demyelination, iDSC may provide a turning point of paranodal demyelination becoming segmental demyelination

A new diagnostic definition of peripheral neuropathy, called nodo/paranodopathy, has been used beyond the classical definition of axonopathy or demyelinopathy [41]. Nodo/paranodopathy encompasses the neuropathy by autoantibodies to the ganglioside of the node in the axonal form of GBS, acute motor axonal neuropathy, or by the loss of nodal function due to autoantibodies against junctional proteins in nodal/paranodal areas, such as gliomedin, cortactin1, and neurofascin [41, 47–51] (Fig. 4). The phenomenon of reversible conduction block (RCF) represents the transient failure of the development of action potentials caused by the dysfunction of sodium or potassium channels in the node region by these autoantibodies [41]. Koike et al. recently showed that macrophages and segmental demyelination were not observed in CIDP with an anti-NF155 antibody, suggesting that the autoantibody-induced CIDP corresponds to the macrophage-independent demyelinating neuropathy [50], which constitutes approximately 10–20% of CIDP patients. From a pathological viewpoint, electron microscopic observations of the patient’s sural nerves of the CIDP with nodal autoantibodies revealed that the space between the paranodal loop and axonal membrane was abnormally extended, which reflected RCF or paranodal attack by the antibodies without signs of MAD [50]. Therefore, this pathological phenomenon suggests that inflammatory demyelinating neuropathy caused by a specific autoantibody against a node or paranode does not sufficiently induce iDSC and preclude the development of segmental demyelination by macrophages (Fig. 4). However, macrophage infiltration into iDSCs would result in the complete elimination of myelin, which shows typical demyelination electrophysiology, i.e., the spreading and latency increase of compound action potentials [41].

Notably, the representative paranodal antibodies, the anti-NF155 and anti-contactin1, are isotype IgG4, and CIDP associated with these antibodies share similarities in not responding well to IVIg treatment [23, 41, 49, 50]. IgG4 is an antibody that does not activate complements, which crucially provokes macrophage activation, and this type of antibody may be less able to activate macrophages for demyelination and induce iDSC transformation. In contrast, macrophage-mediated demyelination in typical GBS and in a subset of CIDP is associated with complement deposition on SC membranes [52, 53], and moreover IVIg treatment reduced the infiltration of macrophages and induced a relief of symptoms in the EAN model [54]. Therefore, the presence of iDSC-MAD cooperation may underlie the differential responses of IVIg treatment in a large spectrum of CIDP pathogenesis.

## Inflammatory demyelinating Schwann cells may provide internodal routes for macrophage invasion

**Fig. 5:**
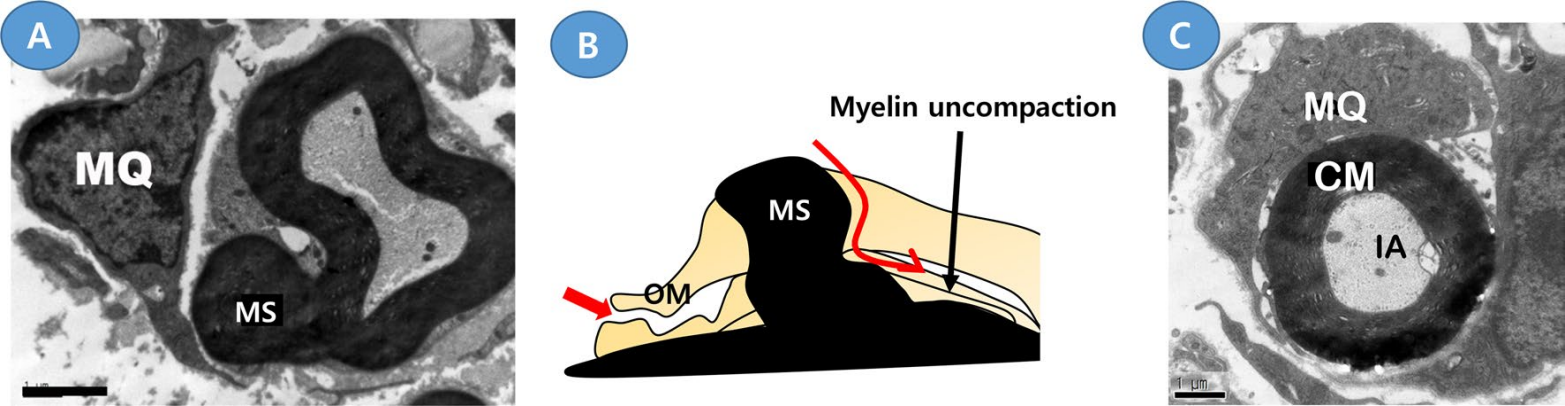
Potential internodal routes for macrophage invasion in a demyelinating Schwann cell in inflammatory demyelination. Two electron microscopies were obtained from the sciatic nerves of B7-2/NOD inflammatory demyelinating neuropathy mice [10, 12]. **a** A transmission electron microscopy shows the generation of a myelinosome (MS) from the main myelin sheath. **b** Schematic drawing showing two potential internodal routes for macrophage invasion (red arrows). *OM* the outer mesaxon. **c** A transmission electron microscopy shows a macrophage (MQ) encircles the compact myelin (CM), which contains an intact axon (IA) but is separated from the abaxonal cytoplasm of the Schwann cell

Myelin uncompaction in iDSC is accompanied by myelin outfolding or myelin clump formation [12, 27] (Fig. 5a), and the clump finally becomes a myelinosome, which is an isolated degenerated myelin globule in the hypertrophic cytoplasm. The separation of myelinosomes from the main myelin sheath requires the cleavage and repair of the myelin membrane, which similarly occur during myelin fragmentation in wDSC. The high levels of lysosomal enzymes around myelinosomes indicate a self-digestion of the myelinosome by iDSCs, and we previously reported the induction of autolysosomes in inflammatory demyelinating neuropathy [12]. This myelinosome was fused with the outer plasma membrane of iDSCs, and thus the degenerating myelins were exposed to the basement membrane of the endoneurium [10], in a manner similar to wDSCs, and this exposure may provide a route for macrophage invasion into the myelin uncompaction area, where the myelinosome originates, within the cytoplasm of iDSCs (Fig. 5a, b).

Previous electron microscopy of the neuropathic nerves of rat EAN revealed a mesaxonal gap that reflected the degeneration of the outer mesaxon [26](Fig. 5b). This region also has a structure of adherens junction composed of E-cadherin complex [38], and the selective destruction of E-cadherin in the EAN and B7-2 knockout CIDP models supported the structural modification of the outer mesaxon [12]. A degenerated outer mesaxon may be an infiltration site for macrophages [26], and this degeneration of the outer mesaxon is radially connected to myelin uncompaction regions, which may allow the infiltrated macrophages to intrude through the mesaxon into the deep side of the SC cytoplasm and then the macrophage finally resides outside of the compact myelin, separating it from its generating SC [11, 26] (Fig. 5b, c). Taken together, the infiltration of macrophages through the internode might be also assisted by the acquisition of the myelin clearance ability of SCs in response to immune attacks.

In conclusion, the segmental demyelination in inflammatory demyelinating neuropathy is completed via the digestion of myelin by macrophages. However, it appears to rely on the assistance of iDSCs, which would provide several potential routes for macrophage invasion into the SC cytoplasm. Although specific biochemical mechanisms, such as a region-specific epitope allowing macrophage invasion at a specific locus, cannot be completely excluded, the cellular mechanisms by which macrophages invade for myelin digestion within a living SC may be explained by introduction of the concept of iDSCs. Therefore, future mechanistic studies of SC plasticity will be important to understand the mechanisms of macrophage-dependent demyelination and the development of therapeutic strategies for inflammatory demyelinating neuropathies.
